# Association between systemic inflammatory indices, neuro-COVID manifestations, and in-hospital death: a retrospective study

**DOI:** 10.25122/jml-2026-0134

**Published:** 2026-09

**Authors:** Iulia Elena Diaconu, Maria Ioana Onofrei, Raluca Erika Irimie-Baluta, Andrei Nicolae Avădanei, Gabriela Lacramioara Juganariu, Ioana Alina Harja-Alexa, Andrei Vâță, Mihaela Catalina Luca

**Affiliations:** 1 Doctoral School, Grigore T. Popa University of Medicine and Pharmacy, Iasi, Romania; 2 Department of Infectious Diseases, Grigore T. Popa University of Medicine and Pharmacy, Iasi, Romania; 3 Department of Infectious Diseases, Sf. Parascheva Clinical Hospital of Infectious Diseases, Iasi, Romania; 4 Department of Vascular Surgery, Sf. Spiridon Emergency Hospital, Iasi, Romania

**Keywords:** encephalopathy, COVID-19, neutrophil-to-lymphocyte ratio, C-reactive protein-to-lymphocyte ratio, hospital mortality, AUC, area under the curve, CI, confidence interval, CLR, C-reactive protein-to-lymphocyte ratio, COVID-19, coronavirus disease 2019, CRP, C-reactive protein, ICU, intensive care unit, IQR, interquartile range, NLR, neutrophil-to-lymphocyte ratio, OR, odds ratio, qRT-PCR, quantitative reverse transcription polymerase chain reaction, ROC, receiver operating characteristic, SARS-CoV-2, severe acute respiratory syndrome coronavirus 2, VOC, variant of concern, WBC, white blood cells, WHO, World Health Organization

## Abstract

Neurological manifestations are frequently reported among hospitalized patients with coronavirus disease 2019 (COVID-19), but their temporal distribution and prognostic relevance may vary across pandemic periods dominated by different severe acute respiratory syndrome coronavirus 2 (SARS-CoV-2) variants of concern (VOCs). This single-center retrospective study assessed neurological syndromes and prognostic factors in 496 unvaccinated hospitalized patients with mild-to-moderate SARS-CoV-2 infection presenting with neurological manifestations at admission. Patients were grouped by pandemic period, defined by the predominant VOC (group 1, pre-Delta, *n* = 173; group 2, Delta, *n* = 209; group 3, Omicron, *n* = 114). Neurological manifestations and systemic inflammatory indices, including the C-reactive protein-to-lymphocyte ratio (CLR) and the neutrophil-to-lymphocyte ratio (NLR), were evaluated in relation to in-hospital death. The frequency of encephalopathy differed significantly across the periods analyzed and was highest in group 2 compared with groups 1 and 3 (44.50% vs 24.28% and 31.58%, respectively; *P* < 0.001 and *P* = 0.023, respectively), with no significant difference between groups 1 and 3. Among patients with severe COVID-19 who required intensive care unit admission during hospitalization, CLR and NLR were higher in group 2 (Delta predominance). Elevated NLR was associated with death in group 1 (odds ratio [OR] = 1.778; 95% confidence interval [CI], 1.225–5.136; *P* = 0.010), group 2 (OR = 12.567; 95% CI, 3.109–18.766; *P* = 0.025), and group 3 (OR = 2.222; 95% CI, 1.373–4.080; *P* = 0.003). Elevated CLR was also associated with death in group 1 (OR = 6.938; 95% CI, 2.043–12.156; *P* = 0.021), group 2 (OR = 6.536; 95% CI, 5.711–21.221; *P* = 0.015), and group 3 (OR = 5.16; 95% CI, 1.865–12.362; *P* = 0.041). The relatively small sample sizes in this study may have limited the statistical power to detect certain associations and contributed to the wide confidence intervals observed by some predictors. Identification of accessible clinical and laboratory predictors of adverse outcomes can support timely intervention and efficient resource allocation in real-world practice with limited resources.

## INTRODUCTION

The coronavirus disease 2019 (COVID-19) pandemic, caused by severe acute respiratory syndrome coronavirus 2 (SARS-CoV-2), has had a substantial impact on global health systems since December 2019. Although COVID-19 was initially regarded as a respiratory illness, there is evidence that SARS-CoV-2 exhibits neurotropism and can affect both the central and peripheral nervous systems. Neurological symptoms are recognized as notable clinical features of COVID-19, contributing to morbidity and mortality. Systematic reviews and meta-analyses have shown a substantial burden of neurological involvement in patients with COVID-19: fatigue occurred in 32% of patients, myalgia in 20%, taste impairment in 21%, smell impairment in 19%, and headache in 13% [1]. Neurological manifestations also include serious central nervous system complications such as encephalopathy, altered consciousness, seizures, and cerebrovascular events [2]. Less common peripheral nervous system manifestations include Guillain–Barré syndrome, which occurred in about 0.04% of cases but was significantly more frequent than in patients without COVID-19 [3]. Neurological manifestations are associated with disease severity and adverse outcomes. Liotta *et al*. observed neurological symptoms at COVID-19 onset in 42.2% of patients, at hospitalization in 62.7%, and at any point in 82.3% of hospitalized patients [4].

The COVID-19 pandemic has progressed through waves driven by SARS-CoV-2 variants of concern (VOCs), including Alpha (B.1.1.7), Beta (B.1.351), Gamma (P.1), Delta (B.1.617.2), and Omicron (B.1.1.529), as classified by the World Health Organization (WHO) [5]. Each variant has distinct epidemiological and clinical characteristics affecting transmissibility, severity, and symptoms. The Delta variant, dominant globally in mid-2021, was linked to greater disease severity than earlier strains; Delta infection increased the risk of hospital admission by 85% compared with Alpha [6,7]. In contrast, the Omicron variant, which emerged in late 2021 and quickly became globally dominant, showed reduced respiratory disease severity but altered clinical profiles. Omicron infection was associated with a 61% lower risk of death and a 56% lower risk of hospitalization compared with Delta [7,8].

In this single-center cohort of hospitalized patients with SARS-CoV-2 infection and neurological symptoms at admission, we aimed to evaluate the distribution of neurological manifestations across three pandemic periods and to identify clinical and laboratory predictors of mortality in this population.

## MATERIAL AND METHODS

This single-center retrospective study was performed at the Sf. Parascheva Clinical Hospital of Infectious Diseases, Iasi, Romania. Data were collected from 1 March 2020 to 31 August 2022.

### Study patients

The study included hospitalized patients aged ≥18 years at admission with mild-to-moderate COVID-19 (score 4–5 on the WHO clinical progression scale), laboratory-confirmed by quantitative reverse transcription polymerase chain reaction (qRT-PCR) on nasopharyngeal/oropharyngeal swab samples, and with documentation in the medical record of at least one new-onset neurological symptom or sign (neuro-COVID-19) at admission (e.g., new-onset or significantly worsened headache, dizziness or vertigo, anosmia or ageusia, seizures, encephalopathy, confusion, or paresthesia). Only unvaccinated patients were selected, to ensure balance between groups and avoid confounding. Encephalopathy was defined in accordance with the Global COVID-19 Neuro Research Coalition: a diagnosis of COVID-19 encephalopathy requires confirmed COVID-19 infection, symptom onset corresponding with the infection, exclusion of alternative diagnoses, and a distinction between direct brain involvement and secondary dysfunction due to illness severity [9]. Patients with moderate-to-severe neuro-COVID-19 (WHO score ≥6 in patients with neurological manifestations at admission) and patients without neurological disturbances at admission were excluded. Patients admitted primarily for another reason, in whom COVID-19 was an incidental finding with no acute COVID-19-related clinical deterioration, and records with missing data were also excluded. For patients with multiple hospitalizations, only the first COVID-19 hospitalization was included. Patients were divided into three groups, each corresponding to a distinct period of SARS-CoV-2 variant predominance based on national surveillance data: group 1, pre-Delta VOCs (March 2020–June 2021); group 2, Delta VOC (July–December 2021); and group 3, Omicron VOC (January–August 2022) [10].

### Data collected

Data were collected retrospectively from the electronic medical records and included demographic data (age, sex), medical and medication history, SARS-CoV-2 infection history (severity of infection, clinical manifestations, treatment, outcome), and laboratory data (full blood count, biochemistry, inflammatory markers). COVID-19 severity was graded on the 11-point WHO clinical progression scale [11]. Complete blood counts were performed using XS-1000i hematology analyzers (Sysmex, Hamburg, Germany), and biochemical parameters were measured using the Rx Imola analyzer (Randox, Antrim, UK). The following systemic inflammatory indices were calculated: the neutrophil-to-lymphocyte ratio (NLR) and the C-reactive protein (CRP; mg/dL)-to-lymphocyte (×10^3^/mL) ratio (CLR) [12].

### Statistical analysis

The primary outcome was in-hospital mortality. Because continuous variables were non-normally distributed, they are presented as medians and interquartile ranges (IQRs) and were compared across the three pandemic-period groups using the Kruskal–Wallis test. Categorical variables are presented as counts and percentages and were compared using the chi-squared test or Fisher’s exact test, as appropriate. Pairwise comparisons were performed after a significant global test and adjusted for multiple comparisons. Multivariable logistic regression models were fitted separately for each pandemic-period group. Given the limited number of deaths and the collinearity between the inflammatory ratios and their component variables, NLR and CLR were evaluated in separate parsimonious models that also included encephalopathy, duration of hospitalization, CRP, white blood cells (WBC), lymphocytes, and platelets. Adjusted odds ratios (ORs) with 95% confidence intervals (CIs) were reported. Model discrimination was evaluated using receiver operating characteristic (ROC) curves and the DeLong test, which compared the areas under the curve (AUC) with sensitivity and specificity. All tests were two-sided, and *P* < 0.05 was considered statistically significant, with adjusted *P* values reported for multiple pairwise comparisons. Analyses were performed using XLSTAT (Lumivero, 2024; XLSTAT statistical and data analysis solution, Paris, France).

## RESULTS

Of 2365 patients with neurological manifestations at admission identified in the hospital database, 496 patients with confirmed neuro-COVID-19 at admission and a complete dataset were included in this analysis (173 in group 1, 209 in group 2, and 114 in group 3). Only unvaccinated patients were included to ensure balance between the study groups (Figure 1).

**Figure 1:**
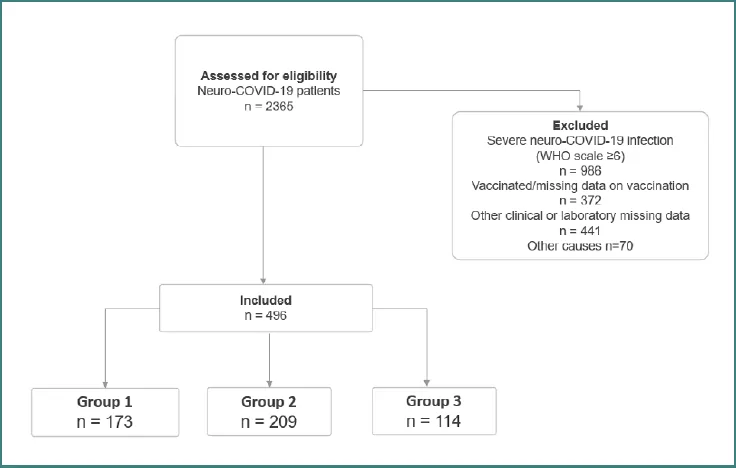
Study flowchart. Patient selection and allocation to the three pandemic-period groups. WHO, World Health Organization.

The median age ranged from 67 to 74 years, with no statistically significant differences among the groups. Sex distribution was also comparable between groups (Table 1). Cardiovascular comorbidities were the most frequent (42.77%–57.02%), followed by obesity (21.93% in group 3 to 23.44% in group 2) and diabetes mellitus (15.61% in group 1 to 19.14% in group 2). COVID-19 severity differed significantly across the periods analyzed. During the pre-Delta (group 1) and Omicron (group 3) periods, most patients did not require oxygen supplementation (66.47% [*n* = 115] in group 1 and 79.82% [*n* = 91] in group 3), compared with only 35.41% (*n* = 74) in group 2 (Table 1).

**Table 1:** Clinical and laboratory characteristics at admission and outcome, by study group

Variable	Group 1 (*n* = 173)	Group 2 (*n* = 209)	Group 3 (*n* = 114)	*P* (G1 vs G2)	*P* (G2 vs G3)	*P* (G1 vs G3)
Age (years), median (IQR)	67 (56–75)	74 (56–79)	67.5 (56–76)	0.672	0.743	0.815
**Sex, %**						
Male	60.69	54.55	53.51	0.227	0.864	0.886
**COVID-19 severity at admission, %**						
WHO score 4 (no oxygen therapy)	66.47	35.41	79.82	**< 0.001**	**< 0.001**	0.192
**Comorbidities at admission, %**						
Cardiovascular	42.77	44.50	57.02	0.734	**0.032**	**0.023**
Neurologic	10.98	12.44	19.30	0.66	0.05	**0.046**
Oncologic	6.36	6.70	12.28	0.893	0.08	0.092
Diabetes mellitus	15.61	19.14	18.42	0.367	0.533	0.517
Obesity	22.54	23.44	21.93	0.835	0.758	0.903
Autoimmune disorders	9.25	5.26	11.40	0.130	**0.045**	0.553
**Laboratory parameters and inflammatory markers at admission, median (IQR)**						
CLR	65.6 (17.1–134.9)	62.3 (16.0–203.1)	63.6 (16.3–137.7)	0.39	0.49	0.751
NLR	5.48 (2.60–10.45)	4.14 (2.46–8.45)	5.39 (2.68–10.36)	0.30	0.64	0.710
CRP (mg/L)	67.94 (19.92–112.70)	75.93 (13.83–183.58)	85.09 (24.79–136.80)	0.48	0.65	0.070
**COVID-19 outcome, %**						
Death	6.94	12.44	7.89	0.074	0.209	0.762

Data are presented as median (IQR) or percentage of patients. Continuous variables were compared with the Kruskal-Wallis test and categorical variables with the chi-squared test or Fisher's exact test, as appropriate; the *P* values shown are for pairwise comparisons adjusted for multiple comparisons. Bold *P* values indicate statistical significance (*P* < 0.05). COVID-19 severity was graded on the 11-point WHO clinical progression scale (score 4, hospitalized, no oxygen therapy). CLR, C-reactive protein-to-lymphocyte ratio; COVID-19, coronavirus disease 2019; CRP, C-reactive protein; G1/G2/G3, group 1/2/3; IQR, interquartile range; NLR, neutrophil-to-lymphocyte ratio; WHO, World Health Organization.

### Outcomes

The death rate was low in all three groups: 6.94% (12 patients) in group 1, 12.44% (26 patients) in group 2, and 7.89% (9 patients) in group 3. No significant differences in death rates were found between groups (Table 1).

### COVID-19-related neurological manifestations

The neurological manifestations identified at admission in all three groups were headache, fatigue, dizziness, anosmia, ageusia, confusion, and encephalopathy (Figure 2). Headache was the most commonly reported neurological symptom, with a slightly higher frequency in group 3 (78.95%) than in group 1 (68.79%) (*P* = 0.058).

**Figure 2:**
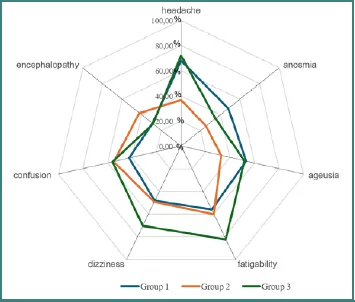
Neurological manifestations at admission, by study group. Radar chart showing the frequency of each manifestation (% of patients) in group 1 (pre-Delta period), group 2 (Delta period), and group 3 (Omicron period).

The frequency of encephalopathy was highest in group 2 (44.50%, *n* = 93) compared with group 1 (24.28%, *n* = 42; *P* < 0.001) and group 3 (31.58%, *n* = 36; *P* = 0.023), with no significant difference between groups 1 and 3 (*P* = 0.174) (Figure 2).

### Inflammatory markers

NLR and CLR values at admission did not differ significantly between groups (Table 1). A subgroup analysis of patients who required intensive care unit (ICU) admission during hospitalization (21 patients in group 1, 54 in group 2, and 12 in group 3) showed significantly higher values at admission in group 2 (Delta VOC) than in groups 1 and 3 for NLR (median, 5.676 [IQR, 2.63–9.76] in group 1; 6.123 [IQR, 4.14–13.14] in group 2; and 3.73 [IQR, 2.92–6.34] in group 3; *P* = 0.011 for group 1 vs group 2, *P* = 0.035 for group 2 vs group 3, and *P* = 0.871 for group 1 vs group 3) and CLR (median, 61.99 [IQR, 19.826–120.625] in group 1; 71.310 [IQR, 32.49–246.055] in group 2; and 69.089 [IQR, 26.24–126.32] in group 3; *P* < 0.001 for group 1 vs group 2, *P* < 0.001 for group 2 vs group 3, and *P* = 0.280 for group 1 vs group 3) (Figure 3).

**Figure 3:**
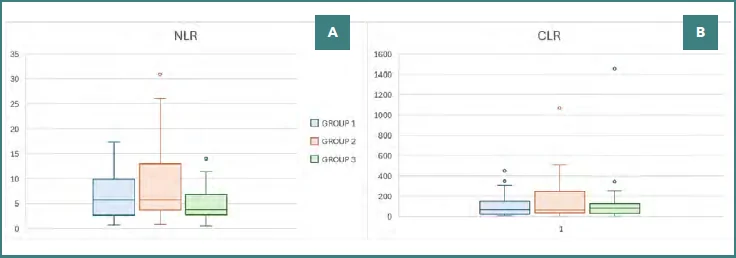
NLR and CLR at admission in patients who required ICU admission, by study group. (A) NLR; (B) CLR. CLR, C-reactive protein-to-lymphocyte ratio; ICU, intensive care unit; NLR, neutrophil-to-lymphocyte ratio.

### Analysis of risk factors for negative outcomes

Several of the analyzed variables were significantly associated with mortality across the study groups. Among the neurological manifestations, encephalopathy was significantly associated with mortality in all three groups (OR = 2.180; 95% CI, 1.408–11.643 in group 1; OR = 3.717; 95% CI, 1.775–7.786 in group 2; and OR = 2.064; 95% CI, 1.031–4.133 in group 3) (Table 2).

**Table 2:** Multivariable logistic regression for predictors of in-hospital death, by study group

	Group 1 (*n* = 173)			Group 2 (*n* = 209)			Group 3 (*n* = 114)		
Variable	OR	95% CI	*P*	OR	95% CI	*P*	OR	95% CI	*P*
Duration of hospitalization (days)	0.976	0.919–1.036	0.131	1.010	0.873–1.389	0.393	0.935	0.880–1.994	0.346
Encephalopathy (present vs absent)	2.180	1.408–11.643	0.039	3.717	1.775–7.786	0.022	2.064	1.031–4.133	0.033
WBC (×10^3^/mL)	1.003	0.712–1.413	0.985	1.173	0.195–1.443	0.395	0.585	0.285–1.201	0.142
Lymphocytes (×10^3^/mL)	0.756	0.341–1.124	0.867	1.229	0.280–1.586	0.146	0.993	0.911–1.084	0.502
Platelets (×10^3^/mL)	0.171	0.070–1.027	0.230	0.845	0.773–1.924	0.157	0.449	0.171–1.179	0.142
CRP (mg/L)	1.004	0.996–1.012	0.294	1.051	0.532–2.074	0.433	1.007	0.004–1.022	0.485
NLR	1.778	1.225–5.136	0.010	12.567	3.109–18.766	0.025	2.222	1.373–4.080	0.003
CLR	6.938	2.043–12.156	0.021	6.536	5.711–21.221	0.015	5.162	1.865–12.362	0.041

Adjusted ORs from multivariable logistic regression models fitted separately for each study group; outcome, in-hospital death. NLR and CLR were entered in two separate models. CI, confidence interval; CLR, C-reactive protein-to-lymphocyte ratio; CRP, C-reactive protein; NLR, neutrophil-to-lymphocyte ratio; OR, odds ratio; WBC, white blood cells.

Logistic regression showed that WBC, lymphocytes, platelets, and CRP were not associated with the outcome. In contrast, NLR and CLR were associated with death in all three study groups. The highest risk was identified in group 2 (July–December 2021) for NLR (OR = 12.567; 95% CI, 3.109–18.766) and in group 1 for CLR (OR = 6.536; 95% CI, 5.711–21.221).

ROC curve analysis was performed to assess the discriminatory ability of NLR and CLR for mortality within groups (Table 3).

**Table 3:** Discriminatory performance of NLR and CLR for in-hospital death: AUC, sensitivity, and specificity

Variable	Group	AUC	Sensitivity	Specificity	*P*
NLR	Group 1	0.755	0.762	0.645	**0.001**
	Group 2	0.857	0.727	0.857	**< 0.001**
	Group 3	0.684	0.714	0.550	**0.008**
CLR	Group 1	0.661	0.714	0.545	**0.018**
	Group 2	0.753	0.731	0.568	**< 0.001**
	Group 3	0.612	0.607	0.544	0.083

Receiver operating characteristic curve analysis using DeLong test; sensitivity and specificity are given as proportions at the optimal cutoff. Bold *P* values indicate statistical significance (*P* < 0.05). AUC, area under the curve; CLR, C-reactive protein-to-lymphocyte ratio; NLR, neutrophil-to-lymphocyte ratio.

For NLR, the optimal cutoff was 3.596 (AUC = 0.755; sensitivity, 0.762; specificity, 0.645) in group 1, 4.409 (AUC = 0.857; sensitivity, 0.727; specificity, 0.857) in group 2, and 5.409 (AUC = 0.684; sensitivity, 0.714; specificity, 0.550) in group 3 (Table 3).

For CLR, the optimal cutoff was 65.62 (AUC = 0.753; sensitivity, 0.731; specificity, 0.568) in group 2. Because the cutoffs were derived and evaluated in the same dataset, their clinical performance may be optimistic and requires external validation.

## DISCUSSION

This retrospective study of 496 patients with SARS-CoV-2 infection and neurological manifestations across three pandemic periods identified significant differences in the distribution of neurological symptoms, disease severity, and prognostic factors. This temporal classification reflects periods of variant dominance rather than individually confirmed viral genotypes; observed differences between groups may therefore reflect changes in clinical management, healthcare capacity, population immunity, or vaccination status, rather than intrinsic properties of specific variants. Only unvaccinated patients were included in this study to maintain balance across the three groups (an extended part of the pre-Delta period was not covered by vaccination in Romania). Significantly higher disease severity was observed during the Delta-predominant period (group 2), with a higher proportion of patients requiring oxygen supplementation. As severity differences across pandemic periods may act as a confounder or mediator of this association, a causal attribution to variant-specific properties cannot be established in our retrospective study. Brola and Wilski noted a high risk of neuropsychiatric complications and death with Delta, including increased cognitive deficits, epilepsy, and ischemic stroke [13]. Taquet *et al*. documented that the risks of ischemic stroke, epilepsy, cognitive deficits, insomnia, and anxiety disorders were similar for the Delta, Alpha, and Omicron VOCs [14]. In our study, encephalopathy was more frequent in group 2 (44.50%) than in group 1 (24.28%) and group 3 (31.58%). Kulkarni *et al*. reported that encephalopathy (34.5%) was the most common neurological manifestation, particularly in the first wave [15]. The increased frequency of encephalopathy during the Delta period may reflect a greater propensity of this variant to induce severe systemic inflammation and cytokine dysregulation, resulting in toxic-metabolic disturbances and central nervous system injury [16,17]. Specific neurological manifestations, particularly encephalopathy (ORs of 2.180 in group 1, 3.717 in group 2, and 2.064 in group 3), independently predicted mortality in the groups analyzed. Di Giorgio *et al*. reported that delirium was the strongest independent predictor of death or ICU admission, with patients with delirium having an almost fourfold probability of a negative outcome (hazard ratio 3.81 for any delirium type and 5.95 for hypoactive delirium) [18]. Corona-Nakamura *et al*. found that specific neurological symptoms, including delirium (OR = 4.51), altered consciousness (OR = 10.41), and stupor (OR = 8.02), were significant predictors of mortality in patients with COVID-19 and neurological manifestations [17].

Multivariable logistic regression analysis indicated that the systemic inflammatory indices NLR (ORs of 1.778 in group 1, 12.567 in group 2, and 2.222 in group 3) and CLR (ORs of 6.938 in group 1, 6.536 in group 2, and 5.16 in group 3) were independently associated with death in all three pandemic periods analyzed. These findings align with extensive literature documenting the prognostic significance of these inflammatory markers in COVID-19. Parthasarathi *et al*. conducted a systematic review and meta-analysis demonstrating that NLR on admission predicts both severity and mortality in patients with COVID-19, with an NLR > 6.5 associated with significantly greater odds of mortality [19]. Regolo *et al*. found that an NLR cutoff of 11.38 showed 72.9% sensitivity and 71.9% specificity for mortality, with an AUC of 0.772 [20]. A large meta-analysis reported pooled sensitivity and specificity for mortality of 0.80 and 0.78, respectively, with an optimal NLR cutoff of ≥6 for mortality prediction [21]. Elevated CLR is a reliable marker of poor COVID-19 outcomes; a cutoff of 78.3 (sensitivity, 79%; specificity, 47%) has been identified as a predictor of COVID-19 severity, and a cutoff of 159.5 (sensitivity, 48%; specificity, 70%) as a predictor of mortality [22].

These results could have important implications for early risk stratification in patients with COVID-19. Identification of accessible clinical and laboratory predictors of adverse outcomes can support timely intervention and efficient resource allocation. Comprehensive risk assessment should incorporate demographic factors, comorbidity burden, inflammatory biomarkers (such as NLR and CLR), and specific neurological symptoms, especially encephalopathy [23]. Timely identification of high-risk patients enables earlier implementation of targeted isolation, monitoring, and therapeutic measures, improving patient outcomes.

Several limitations of this study warrant consideration. First, the retrospective design introduces potential for selection bias and limits the ability to establish causal relationships between predictors and outcomes. Second, as a single-center study, our findings may not be fully generalizable to other healthcare settings with different patient populations, resource availability, or clinical practices. Third, the relatively small sample sizes may have limited the statistical power to detect certain associations and contributed to the wide confidence intervals observed for some predictors. Fourth, the classification of patients was based on nationally reported periods of variant dominance rather than individual viral sequencing. Fifth, the definition and assessment of neurological manifestations relied on clinical documentation, which may have varied in completeness and standardization across clinicians and time periods. Despite these limitations, our study provides valuable real-world data on the evolving clinical profile of COVID-19-related neurological manifestations and identifies multiple predictors of adverse outcomes to inform risk stratification and clinical management.

## CONCLUSION

This retrospective study demonstrates significant differences in clinical presentation and disease severity across three pandemic periods characterized by the predominance of different SARS-CoV-2 VOCs. Our findings identified clinical, laboratory, and neurological parameters that are independently associated with mortality in patients with COVID-19 and may represent candidate predictors for formal risk stratification models. However, prospective validation in independent cohorts is required before any of these factors can be recommended as components of a clinical decision-making tool.

## Data Availability

The data supporting the findings of this study are available from the corresponding author upon reasonable request.
